# Language for Winning Hearts and Minds: Verb Aspect in U.S. Presidential Campaign Speeches for Engaging Emotion

**DOI:** 10.3389/fpsyg.2016.00899

**Published:** 2016-06-22

**Authors:** David A. Havas, Christopher B. Chapp

**Affiliations:** ^1^Department of Psychology, University of Wisconsin-Whitewater, WhitewaterWI, USA; ^2^Department of Political Science, St. Olaf College, NorthfieldMN, USA

**Keywords:** language, emotion, embodied cognition, rhetoric, syntax, alignment

## Abstract

How does language influence the emotions and actions of large audiences? Functionally, emotions help address environmental uncertainty by constraining the body to support adaptive responses and social coordination. We propose emotions provide a similar function in language processing by constraining the mental simulation of language content to facilitate comprehension, and to foster alignment of mental states in message recipients. Consequently, we predicted that emotion-inducing language should be found in speeches specifically designed to create audience alignment – stump speeches of United States presidential candidates. We focused on phrases in the past imperfective verb aspect (“a bad economy was burdening us”) that leave a mental simulation of the language content open-ended, and thus unconstrained, relative to past perfective sentences (“we were burdened by a bad economy”). As predicted, imperfective phrases appeared more frequently in stump versus comparison speeches, relative to perfective phrases. In a subsequent experiment, participants rated phrases from presidential speeches as more emotionally intense when written in the imperfective aspect compared to the same phrases written in the perfective aspect, particularly for sentences perceived as negative in valence. These findings are consistent with the notion that emotions have a role in constraining the comprehension of language, a role that may be used in communication with large audiences.

## Introduction

Language causes powerful and reliable changes in the emotions and actions of large audiences, as when a skillful politician rallies voters to the polls (Brader, 2006; Chapp, 2012). How does language interact with emotion to affect the behaviors of large audiences? Emotion theorists suggest that a fundamental function of emotion is to prioritize some actions over others in order to support adaptive responses to real world challenges (Frijda, 1986; Keltner and Gross, 1999; Levenson, 2003; Norman et al., 2014) and to promote action coordination within social groups (Hatfield et al., 1994; Keltner and Haidt, 1999; Niedenthal and Brauer, 2012). That is, emotions constrain the body in ways that facilitate adaptive, socially coordinated actions. In this article, we present initial evidence that emotions constrain processing of language delivered by skilled politicians for creating social cohesion.

According to embodied theories of cognition, language comprehension involves a process of simulation grounded in neural systems for action, perception, and emotion (Barsalou, 2010; Glenberg et al., 2013). For example, words about kicking engage a simulation grounded in neural systems for kicking (Pulvermüller, 2005; van Elk et al., 2010). Likewise, words that are strongly related to emotions engage a simulation grounded in neural systems for producing and perceiving corresponding emotional expressions (Moseley et al., 2011; Citron, 2012). Thus, one explanation for the emotional power of language is that simulation occurs at the lexical level. The extent to which a speaker is able to elicit an emotional response depends largely on the extent to which the words and combinations of words employed encourage simulation.

Evidence indicates that the emotive consequences of language operate above the lexical (individual word) level (Havas et al., 2007, 2010). For example, Havas et al. (2007) measured reading times for sentences describing pleasant or unpleasant situations while participants were in a matching or mismatching emotional state. Although the sentences were emotional, they made little or no reference to emotional states. An example pleasant sentence is, “You can tell you’re executing the complex dive flawlessly.” An unpleasant sentence is “The police car rapidly pulls up behind you, siren blaring.” To covertly manipulate emotional state, they used the procedure of Strack et al. (1988) which reliably influences positive and negative emotional experience in the absence of awareness. Participants held a pen in the teeth to produce a smile, or in the lips to produce a frown or pout. As predicted, processing of pleasant sentences was faster when the pen was held in the teeth (producing a smile) than when it was held in the lips (preventing a smile), and vice versa for the time to process unpleasant sentences. In a subsequent study, they employed the pen manipulation in a lexical decision task with words taken from the stimulus sentences in order to test a lexical priming account of their results. The words they use were rated as being “central to the meaning” of the pleasant and unpleasant sentences. Lexical decisions for words were speeded when preceded by semantically associated words, but not by the pen manipulation.

These findings suggest that emotion plays a role in language understanding above the lexical level, perhaps at the level of a situation model (Zwaan and Radvansky, 1998), or in the combinatorial processes involved in language understanding (see also, Lai et al., 2015; Lüdtke and Jacobs, 2015). To account for interactions of emotion in language processing above the lexical level, we have proposed that emotion states encode physiological (e.g., autonomic) constraints of the body that differentially prioritize the simulation of some actions over others, much as physiological constraints influence the preparation of real actions (Havas and Matheson, 2013).

The goal of the present study is to test an embodied account of how language and emotion interact to influence large audiences: the *emotion-constraint-hypothesis*. Just as emotions that emerge during real world challenges constrain the body to support adaptive responses and promote action coordination within groups, emotions that emerge during language comprehension constrain an ensuing mental simulation of actions in order to support language comprehension and promote alignment (Pickering and Garrod, 2004) in the mental states of message audiences. By alignment, we mean similarity in the mental models (Zwaan and Radvansky, 1998) communicators construct to represent the situation conveyed in the language.

If this hypothesis is correct, then we can predict emotion-language interactions where a speaker’s goal is to align the mental states of a large audience for the purpose of social cohesion. In Study 1, we used a corpus analysis to investigate whether political speeches designed to enlist and mobilize the U.S. electorate make more frequent use of language that engages the constraining function of emotion than comparison speeches. In Study 2, we determined whether such language is indeed perceived as more strongly emotional than comparison language.

Support for *emotion-constraint-hypothesis* comes from recent neuroimaging studies that use inter-subject synchronization analysis techniques to compare the time course of neural responses across different participants exposed to the same naturalistic language. Nummenmaa et al. (2014) found that neural similarity among participants was enhanced when they listened to the same emotionally evocative narratives, relative to unemotional narratives. The synchronization was linearly related to listeners’ self-reported emotional state: participants who showed greater similarity in their moment-to-moment emotional ratings of the stories also showed enhanced similarity in the time course of brain activity while listening to the stories. The authors suggest that emotions drive neural synchronization by facilitating participants’ semantic processing of the language. Inter-subject synchrony is also enhanced by powerful political speeches. Schmälzle et al. (2015) examined neural synchrony across time in brains of participants who listened to speeches from German politicians that varied in rhetorical quality. More rhetorically powerful speeches elicited greater neural synchrony across participants, possibly because these speeches also contained more emotional words. While these studies provide strong evidence of emotion constraint, the authors do not offer precisely how language encourages synchronization. It is to this issue that we now turn.

According to the *emotion-constraint-hypothesis*, emotions are likely to be elicited by sentences or phrases that invite a simulation of action but underspecify the particular actions to be simulated (Havas and Matheson, 2013). The basis for this claim lies in affective neuroscience researching showing that key neural structures for coordinating emotional responses (namely, the amygdala, and insula) are sensitive to environmental ambiguity and uncertainty (Whalen, 2007; Singer et al., 2009). For example, the amygdala can be activated by ambiguity, unpredictability, and polysemy in sentence comprehension (Citron and Goldberg, 2014; Shibata et al., 2014; Lai et al., 2015). Although there is abundant research showing that emotional responses influence subsequent cognitive processing (for a review, see Blanchette and Richards, 2010), our hypothesis makes a functional claim that emotions contribute to language comprehension by differentially prioritizing the simulation of some actions over others (Havas et al., 2010; Havas and Matheson, 2013).

To test this hypothesis, we capitalized on the distinction between the past perfective verb aspect, which indicates an action that has happened in the past (“we were burdened by a bad economy”), and the past imperfective verb aspect, which indicates that past action is ongoing or unfinished (“a bad economy was burdening us”). Research has shown that the imperfective aspect tends to evoke more diverse associations (Coll-Florit and Gennari, 2011) and leaves readers’ mental models of described actions unconstrained relative to the perfective aspect (Madden and Zwaan, 2003). Under the *emotion-constraint hypothesis*, imperfective sentences should be more likely to engage audience emotions for guiding mental simulation of actions, and thus instrumental in language designed to align the mental and emotional states of large audiences - namely, political stump speeches.

## Study 1

Campaign stump speeches have been distinguished as a genre of political rhetoric aimed at compelling specific actions from audience members (namely, to turn out and vote for the candidate; Hart, 2002). We compared stump speeches to the State of the Union Address (SOTU), a genre which is similarly focused on policy prescriptions and the office of the presidency, but is closer to an “essay” in structure, more concerned with initiating policy dialog than bringing about specific citizen actions and emotions (Campbell and Jamieson, 2008). Of these two genres, the stump speech is the one in which audience alignment is imperative, and where we expect the imperfective aspect to figure prominently as a means to constrain a simulation of language content. Thus, we expect the imperfective aspect to appear with greater relative frequency in stump speeches than in SOTU speeches

### Method

We created two unique datasets: a set of speech transcripts from 149 candidate stump speeches from the 2012 presidential campaign, and a set of 48 SOTU addresses (1965–2013) to serve as a comparison group. We obtained speech transcripts from 149 candidate stump speeches from the 2012 presidential campaign, as well as a set of 48 State of the Union (SOTU) addresses (1965–2013). We excluded two SOTU addresses from this period that were delivered in written form. We also included five speeches delivered by recently inaugurated presidents to a joint session of congress, though these speeches are not technically SOTU addresses. See Peters (n.d.) for discussion.

To build the stump speech database, campaign appearances first were identified using Obama and Romney’s campaign schedules, regularly updated by Politico (n.d.)^1^ From this list of appearances, we searched two speech transcription services – Federal News Service and Congressional Quarterly Transcriptions – to build an inclusive set of speech transcriptions. Aiming to capture a comprehensive portrait of the 2012 general election campaign, we included every speech delivered between August 12, 2012 (when Paul Ryan was chosen as running mate) and November 5, 2012 (the day before the national presidential election). Each speech transcript was then cleaned to remove notation (“Jeers from audience”) or words not spoken by the candidate (“AUDIENCE: USA! USA!”). State of the Union addresses were identified using the American Presidency Project’s (n.d.) speech database^2^ We included every speech from Johnson’s 1965 State of the Union through Obama’s most recent address. We began with 1965 because this was the first televised evening SOTU.

We created a content analysis “dictionary” tool designed to identify the imperfective aspect. The dictionary was designed to identify *was* and *were + VERB-ing* sentence constructions, as well as negations (*wasn’t/was not* and *weren’t/were not*). We were careful to exclude sentences written in the present perfect aspect where a past event has present consequences, as in “Let me tell you, we have tried that” and “He’s ignored them.” To construct the dictionary, we first developed a corpus of 3342 commonly used verbs by combining Pennebaker’s Linguistic Inquiry and Word Count software (LIWC, Pennebaker et al., 2007) dictionary verbs with others taken from online English verb lists. For an example dictionary^3^ The full list of verbs is available upon request of the authors.

Next, we used the LIWC software to identify instances where one of these verbs was used in the imperfective aspect. LIWC computed an imperfective score for each speech that adjusts for the length of the speech ([imperfective count/total word count] ^∗^100). This approach allows us to compare how stump speeches and SOTU addresses vary with respect to the relative frequency of the imperfective aspect.

### Results

We used a series of OLS regressions to examine the extent to which speech genre (stump vs. SOTU) predicts the imperfective aspect relative to other potential factors such as the individual speakers or their party affiliations (**Table 1**). Model 1 regressed imperfective scores on a dummy variable for genre, Model 2 tested whether the party of the speaker is related to the use of the imperfective aspect, and Model 3 included dummy variables for each candidate to test whether individual differences are driving results. Each model also controlled for the average number of words per sentence for each speech, in the event that more elaborate or complex sentence constructions co-vary with either a particular genre and/or a particular verb aspect.

**Table 1:** Imperfective aspect in political speech.

	Model 1: genre only		Model 2: party controls		Model 3: candidate controls	
Constant	-0.087^∗∗^	(0.020)	-0.247ˆ**	(0.059)	-0.192ˆ**	(0.069)
Genre	0.108^∗∗^	(0.011)	0.128ˆ**	(0.014)	0.116ˆ**	(0.031)
Words per sentence			0.005ˆ*	(0.002)	0.005	(0.002)
Party			0.031ˆ**	(0.009)		
Romney					0.035	(0.043)
Obama					-0.004	(0.041)
W. Bush					0.001	(0.037)
Clinton					0.008	(0.037)
H.W. Bush					0.000	(0.044)
Reagan					0.005	(0.037)
Carter					-0.010	(0.047)
Ford					0.045	(0.047)
Nixon					0.007	(0.044)
						
*R*^2^	0.332		0.379		0.391	


*Entries are unstandardized regression coefficients with standard errors in parentheses. Dependent variable is the imperfective score for each speech. Genre is a dichotomous variable, where 1 = stump speech and 0 = SOTU address. Party is a dichotomous variable where 1 = a Democratic speaker, 2 = a Republican speaker. ^∗^*p* < 0.05; ^∗∗^*p* < 0.01.*

Consistent with predictions, stump speeches invoke the imperfective aspect with significantly more regularity than SOTU speeches. In a SOTU address of average length (5359 words), the model estimates that presidents will deploy the imperfective about 1.5 times. In an average stump speech of only 3178 words, we would expect to find 4.1 imperfective sentences. None of the speaker dummy variables revealed a detectable effect. Speeches that average more words per sentence and Republican affiliation tended to include more imperfective clauses, however, the addition of these variables had little impact on the effect of the genre variable, which remained the most powerful predictor across all three models.

One possible explanation for why imperfective aspect appears with more frequency in stump speeches is that stump speeches contain more past tense constructions in general than SOTU speeches. To check this, we modified our imperfective content analysis tool, changing all “was/were ___-ing” phrases to simple past tense by deleting the “was/were” and adding “___-ed.” For example, instead of scoring a text with the phrase “was apologizing” we now score it for the word “apologized”. Using this method we found that simple past tense verbs occurred at a higher rate in State of the Union Addresses than in stump speeches, *t*(195) = 11.288, *p* < 0.001. This suggests that the higher incidence of past imperfective aspect in stump speeches is not due to the particular verbs included in the content analysis tool, nor is it the case that we observe more imperfective aspect in stump speeches because they tend to utilize past-tense verb constructions more generally.

### Discussion

Presidential candidates’ stump speeches – an important example of language used to create alignment in large audiences – were found to contain a significantly higher proportion of sentences with the past imperfective verb aspect than the SOTU speeches. These differences in speech genre overshadowed all other differences due to factors like words per sentence, individual candidate, or the candidate’s political party, as adding these factors to the model had little or no impact on the contribution of genre.

Results of Study 1 support the *emotion-constraint-hypothesis* that language used to create alignment in mental and affective states of large audiences (U.S. presidential stump speeches) will be more likely to call on the simulation-constraining function of emotion, relative to comparison speeches. We focused our predictions on past imperfective sentences because they compel a prospective simulation of ongoing action without specifying those actions relative to the perfective sentence construction, and thus were presumed to engage the constraining function of emotion. Study 2 was designed to test this presumption by determining whether imperfective sentences, independent of their lexical emotional content, are in fact perceived as more strongly emotional than perfective sentences.

## Study 2

Participants were asked to evaluate perfective and imperfective aspect sentences found in the presidential stump speeches of Study 1 in terms of their emotion strength and their emotional category. To control for non-critical characteristics of the sentences, each sentence was presented in both aspectual forms. We predicted that sentences written in the imperfective aspect would be rated as more strongly emotional (in either a positive or negative valence direction) than the same sentences written in the perfective aspect.

### Method

#### Participants

Participants were individuals who use Amazon’s Mechanical Turk website, a crowd-sourcing Internet marketplace interface. Participants were paid $1 for their participation, and the study was advertised as a not-for-profit study taking approximately 30 min (the actual mean duration was 23 min). Informed consent was obtained through the use of a written statement embedded in the preview page of the study, and participants gave their consent by clicking a button to proceed. The study was approved by the University of Wisconsin–Whitewater Institutional Research Board, and was conducted according to the principles expressed in the Declaration of Helsinki.

Based on a recent comparison of the mental representations induced by imperfective and perfective aspect reporting small sized effects (Cohen’s d≈0.2; Madden and Zwaan, 2003), we estimated needing 150 participants for 80% power. We continued collecting data until we reached this sample size.

#### Sentence Stimuli

We identified a total 534 sentences containing the past imperfective verb aspect and 383 sentences containing the past perfective verb aspect from the stump speech database. As sentence boundaries sometimes contained several phrases, disfluencies, corrections, or repetitions, sentence lengths ranged widely (20 to 470 characters for imperfective and 11 to 602 for perfective). In such cases, we attempted to extract the smallest complete sentence containing the key verb phrase. From among those sentences with only one verb phrase, whose length was within one standard deviation of the mean sentence length, and whose content did not duplicate other already-selected sentences (presidential campaigners tended to be rather consistent in their stump speech content from appearance to appearance), we then randomly selected 25 imperfective and 25 perfective sentences. The average length for the selected sentences was 42 characters for imperfective and 49 for perfective, and this difference was not statistically significant. Sentences made little or no reference to emotions or emotion concepts.

From the 25 perfective and 25 imperfective sentences taken from the presidential stump speeches of Study 1, we wrote a matching 25 imperfective and 25 perfective sentences (respectively) by changing the aspect. Thus, each sentence was represented twice, once in the perfective aspect and once in the imperfective aspect (see **Table 2** for example sentences). A unique yes/no comprehension question was included after each sentence to ensure participants were reading the sentences for understanding. Within each sentence type, there were an equal number of questions that were answered correctly with a “yes” and with a “no” response.

**Table 2:** Example imperfective and perfective sentences.

Imperfective past tense version	Perfective past tense version
*The folks here were holding signs.^∗^*	*The folks here held signs.*
*Electricity was running through my arm.^∗^*	*Electricity ran through my arm.*
*The middleclass was getting hammered.^∗^*	*The middleclass got hammered.*
*He was reading a newspaper article.^∗^*	*He read a newspaper article.*
*They were working really hard.^∗^*	*They worked really hard.*
*She was packing up a birthday package for him.^∗^*	*She packed up a birthday package for him.*
*I was holding that flag and they were singing those words.*	*I held that flag and they sang those words.^∗^*
*And the American people all across this country were responding.*	*And the American people all across this country responded.^∗^*
*We were coming together and were looking for ways to solve the problems.*	*We came together and looked for ways to solve the problems^∗^*
*At its high point, she was employing 200 people.*	*At its high point, she employed 200 people.^∗^*
*He was asking the question about gasoline prices.*	*He asked the question about gasoline prices.^∗^*
*We were trying to do that too, and it wasn’t working very well.*	*We tried to do that too, and it didn’t work very well.*


*Asterisk (^∗^) indicates the sentence’s original aspect format.*

#### Procedure

The final list of one hundred sentences (50 imperfective and 50 perfective) were presented in a random order to participants by computer with instructions to assign each sentence to an emotion category (afraid, angry, anxious, excited, happy, or sad), and to then rate the emotional valence of each sentence using a 5-point Likert-type scale, from strongly negative to strongly positive. To guard against the possibility that responses would reflect speaker emotion and not respondents’ emotional response, survey instructions clearly asked respondents to Rate each sentence according to how effective it is at **giving you an emotional feeling**, either positive or negative. Note: Most of the sentences are not “about” emotions. So, please **rate how the sentence makes you feel**, rather than what you think the sentence is about.”

### Results

Five individuals participated in the experiment twice, and we used only the first set of data from these subjects. Five participants who had extreme error rates (20% or greater) were excluded from analysis bringing the final sample size to 140. The mean error rate for the remaining participants was 3.1%.

Subjects categorized 3.9% of sentences as “afraid”, 6.6% as “angry”, 16.5% as “anxious”, 20.6% as “excited”, 35% as “happy”, and 12% as “sad.” The remaining 5.3% of sentences received no response. Participants were equally likely to assign an emotional category to imperfective sentences (5.2% uncategorized) and perfective sentences (5.3% uncategorized; Pearson’s χ^2^ = 0.082).

We then quantified the amount of agreement in participants’ categorizations using only trials for which participants provided responses on both measures (emotion category and strength) by calculating type C (two-way random) intra-class correlations in SPSS separately for imperfective (*n* = 44) and perfective sentences (*n* = 42). The intra-class correlation analysis treats missing data by deleting cases listwise, and this accounts for the small sample set. Although participants agreed more in their emotional categorizations of imperfective sentences than in their categorizations of perfective sentences, this difference was not statistically significant; Intra-class correlations were 0.909 and 0.837, respectively, *z* = 1.39, *p* = 0.08 (one-tailed).

In order to test whether imperfective sentences were perceived as emotionally stronger than perfective sentences, we recoded participant valence ratings to reflect valence strength (absolute difference from neutral) rather than valence direction, and subjected the resulting group means to a dependent-measures t-test. As predicted, sentences written in imperfective aspect were rated as more emotionally intense (*M* = 0.9857, *SD* = 0.28986) than the same sentences written in the perfective aspect (*M* = 0.9743, *SD* = 0.28615), mean difference = 0.01140, *SD* = 0.06114, SEM = 0.00517, *t*(139) = 2.205, *p* = 0.029, Cohen’s *d* = 0.189, 95% CI = 0.00118–0.02161. A *post hoc* set of seven dependent-measures *t*-tests on each emotion category (including no response) separately revealed no statistically significant differences after accommodating multiple comparisons with a Bonferroni correction (all *p* > 0.007).

We also conducted a *post hoc* test of the effect by valence by coding as “positive” sentences that were categorized by participants as either “happy” or “excited”, and coding as “negative” sentences that were categorized by participants as either “afraid”, “angry”, “anxious”, or “sad”. Disaggregating positive and negative sentences resulted in a larger dataset but with a substantial number of zeroes (44, or 6.9%, with 23 for perfective sentences and 21 for imperfective sentences) making the distribution of absolute value scores non-normal. We therefore excluded these observations before conducting paired samples *t*-tests separately for positive and negative valence sentences. The result was a significant effect of aspect in sentences rated as negative, *t*(139) = 2.497, *p* = 0.014, mean difference = 0.026, but no effect of aspect in sentences rated as positive, *t*(138) = 0.236, *p* = 0.814, mean difference = 0.002.

## General Discussion

Two studies tested the *emotion-constraint-hypothesis*: that emotional responses play a functional role in language processing by constraining mental simulation and thereby promoting alignment in the mental states of large audiences. In Study 1, we found that political rhetoric used to create alignment in the American electorate, U.S. Presidential stump speeches, is more likely than comparison speeches to contain sentences using the past imperfective aspect. Such sentences were hypothesized to call upon the constraining function of emotion relative to perfective sentences because they compel a prospective simulation of ongoing action without specifying those actions (Havas and Matheson, 2013).

In Study 2, we tested the hypothesis that imperfective speech sentences are more likely to engage audience emotions with an emotional rating task. As predicted, participants rated stump speech sentences in the imperfective aspect as more strongly emotional than the same sentences in the perfective aspect. An important qualification of this finding is that the observed effect size is small (Cohen’s *d* = 0.189). However, small effects in the domain of cognition and emotion – particularly in real-world contexts of mass communications – are likely to be meaningful as small changes in emotional language can lead to large-scale shifts in emotional behavior (e.g., Kramer et al., 2014). *Post hoc* tests suggested this effect is somewhat larger when considering only those sentences that participants perceive as having negative valence, perhaps because negative events are associated with a greater number of action response options (Rozin and Rozyman, 2001), and are therefore more open-ended in general, than positive events. However, it’s not yet clear which properties of our stimulus sentences might be critical in this perception. Future research should use validated emotional stimuli to determine whether the effect is limited to a particular emotion or valence category.

These findings extend investigations of emotion-language interactions above the lexical level of processing (e.g., Havas et al., 2007, 2010; Lai et al., 2015). Using a lexical level account, we might predict that sentences in the imperfective aspect appear more frequently in stump speeches because they contain words having closer associations with emotion concepts or emotion states than do perfective sentences. The more emotionally associated imperfective past tense sentences are presumably more effective in compelling audience members to turn out and vote. This type of account is challenged by the results of Study 2 that controlled for lexical content and showed that verb aspect alone is capable of influencing emotional perceptions. Still, it is possible that other systematic lexical differences between types of speeches could influence, or interact with, verb aspect to produce emotional effects beyond those of grammatical aspect. Our data do not discount this, but they further our understanding of how words can be combined at the grammatical level to influence emotion.

Of course, our conclusions are limited by our choice of corpus, and by our methods of sampling. Future studies can explore whether these effects generalize to other contexts and other forms of ambiguous language at syntactic and situation model levels. One salient question is whether our verb phrases produce stronger or weaker effects when processed in isolation than when processed within their speech context. Verb aspect seems to play a pivotal role in situation model construction by modulating the accessibility of text-based information and world knowledge relevant to the text (Madden and Zwaan, 2003; Ferretti et al., 2007). If emotion constraint is involved in this role, then we would predict an enhanced effect for verb phrases when processed as part of a meaningful text. In addition, future research should determine whether such effects might be mediated by embodied emotions as suggested by our previous work (Havas et al., 2007, 2010; Havas and Matheson, 2013). While our predictions were derived from theory and research in embodied cognition, our study provides only indirect support for embodied theories because we did not manipulate or measure the body directly.

Why should the past imperfective grammatical aspect preferentially engage emotion? We sketch an account based on an embodied theory of emotional language comprehension (Havas and Matheson, 2013). Much as emotion constrains the preparation of real actions in order to meet environmental demands (e.g., Frijda, 1986; Barrett, 2006), emotion states differentially prioritize the mental simulation of some actions over others in order to support language comprehension. When effective action is unspecified or underspecified by the language (i.e., the language is ambiguous), there will be a failure to complete a simulation of the sentence content. The function of emotion in such cases is to modulate the action system and influence a simulation. Take the sentence, “Families were struggling to make the mortgage.” Because the imperfective aspect suggests the struggle is ongoing, any emotions associated with struggling will continue to play a role in the ensuing simulation. By contrast, in simulating the perfective “Families struggled to make the mortgage,” the struggle is now over and any emotions associated with struggling need not affect the simulation. Although in both cases the language is emotional, we would predict that the first sentence would lead to greater involvement of emotional activity than the second sentence.

This mechanism complements recent findings of enhanced alignment of neural states in participants exposed to the same emotional narratives and political speeches from neuroimaging studies that have used inter-subject synchronization analysis techniques (e.g., Nummenmaa et al., 2014; Schmälzle et al., 2015). Our use of corpus data reveals one way that alignment effects could be harnessed in real-world settings. It should be noted, however, that our effort to find evidence of enhanced alignment resulting from imperfective aspect in Study 2 was unsuccessful. Future research should aim to directly test the *emotion-constraint-hypothesis* prediction that imperfective aspect fosters alignment in the mental states of an audience by probing the content and degree of similarity among participants’ mental representations. The present data are consistent with this claim but inconclusive.

Our account may shed light on current studies of verb aspect and emotion. Hart (2013) found participants who recounted emotional autobiographical experiences using imperfective aspect experienced stronger corresponding shifts in mood than participants using perfective aspect, and speculated that imperfective aspect enhanced participants’ access to the details of event memory. However, an episodic memory account is unlikely in our study where participants responded to language produced by others. Instead, our results suggest that the open-ended simulation process initiated by the imperfective aspect more effectively draws on emotion for its guiding role in language processing.

Two alternative accounts should be considered, and each is based on the notion that imperfective aspect privileges emotional responding because it describes ongoing actions that persist in time relative to completed events described by perfective aspect. The first suggests that simulating ongoing events draws attention to details about the described event. Indeed, several studies suggest that the imperfective aspect enhances access to discursive detail relative to perfective aspect, including detail about characters (Carreiras et al., 1997), events (Magliano and Schleich, 2000), locations (Ferretti et al., 2007), and visual objects (Madden and Therriault, 2009) that are implied in the language. In these studies participants were provided with a probe word to demonstrate that the probe concept was more cognitively available after reading sentences in the imperfective versus perfective aspect, and thus the probe may have served as a critical constraint needed to complete a simulation. By contrast, in studies that present participants with an open-ended task (more than one probe, or no probe) the imperfective aspect has been found to leave readers’ representations less, not more, constrained relative to perfective aspect (Madden and Zwaan, 2003; Bergen and Wheeler, 2010; Coll-Florit and Gennari, 2011). For example, Coll-Florit and Gennari (2011) showed that reading language about ongoing events leads more diverse semantic associations than language about completed events, a finding that leads us to a second alternative explanation: If ongoing events are associated with a greater diversity of emotional experiences, then our observed emotion advantage for imperfective sentences might be attributable to broader semantic priming of emotion concepts. In such a case, however, participants would have been expected to categorize imperfective sentences with less, not equal or greater, uniformity than perfective sentences.

Finally, our findings add to the research suggesting that the use of verb aspect has political ramifications. Fausey and Matlock (2011) found that describing a positive or negative behavior by a politician using the imperfective aspect (versus perfective aspect) made a larger proportion of participants feel strongly confident that the politician would or would not be reelected, respectively. This may be because imperfective aspect also made participants’ action inferences more extreme (e.g., they estimated that a politician had taken a larger sum of hush money when taking hush money was described using the imperfective aspect). Our findings suggest that audiences viewing a typical stump speech are likely to have a qualitatively different experience than those viewing a SOTU, characterized by more strongly valenced emotions. The mechanism responsible for this difference is emotion constraint, brought about by subtle differences in verb tense.

## Author Contributions

DH and CC contributed equally to the conceptualization and design of the work, the acquisition and analysis of data, the drafting and revising of the manuscript, the final approval of the version to be published, and are equally accountable for all aspects of the work regarding its accuracy and integrity.

## Conflict of Interest Statement

The authors declare that the research was conducted in the absence of any commercial or financial relationships that could be construed as a potential conflict of interest.

## Footnotes

1 http://www.politico.com/2012-election/calendar/.

2 http://www.presidency.ucsb.edu/.

3 http://www.englishclub.com/vocabulary/regular-verbs-list.htm.
